# Knockdown THOC2 suppresses the proliferation and invasion of melanoma

**DOI:** 10.1080/21655979.2019.1685727

**Published:** 2019-11-02

**Authors:** Xiaowei Zhou, Xing Liu, Guoqiang Zhang, Qian Zhang, Hao Chen, Yan Wang, Fang Fang, Jianfang Sun

**Affiliations:** a Jiangsu Key Laboratory of Molecular Biology for Skin Diseases and STIs, Institute of Dermatology, Chinese Academy of Medical Sciences and Peking Union Medical College, Nanjing, P.R.China; b Department of Dermatology, The 1th Hospital of Hebei Medical University, Shijiazhuang, P.R.China

**Keywords:** Melanoma, THOC2, cAMP, microarray

## Abstract

Melanoma is a potentially fatal form of skin cancer with great metastatic potential. THOC2 plays a vital role in human biological progression, however, the roles of THOC2 in melanoma tumorigenesis are still unknown. In the present study, our data demonstrated that THOC2 expression was significantly increased in melanoma tissues, and high THOC2 expression was associated with poor overall survival of melanoma patients. THOC2 reduction repressed melanoma cell proliferation and invasion, and induced cell apoptosis in vitro. Microarray data revealed that the cAMP signaling pathway was significantly downregulated in A375 cells transfected with si-*THOC2*, which was further confirmed by RT-qPCR and bioinformatics analysis. In conclusion, our data indicated that THOC2 might act as an oncogene in melanoma progression through cAMP signaling pathway regulation, which may offer a therapeutic target for melanoma treatment.

## Graphical abstract

**Figure:**
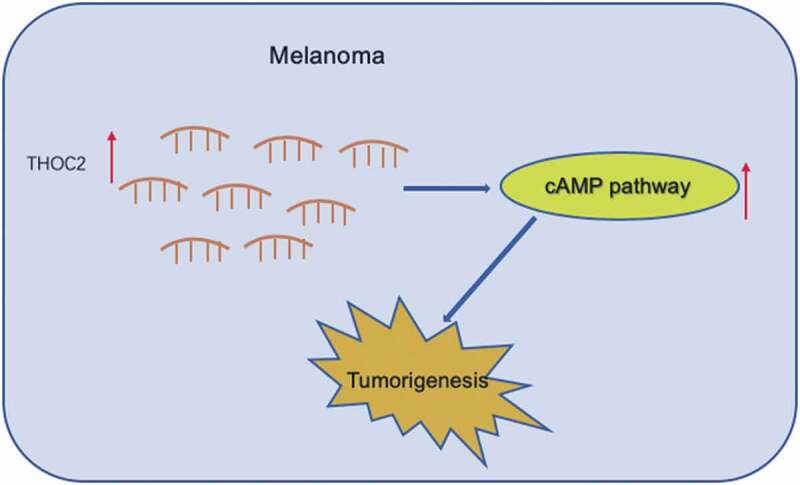

**Abbreviations**: PVDF: polyvinylidene difluoride; MTT: 3-(4,5-dimethylthiazol-2-yl)-2,5-diphenyltetrazolium bromide; SDS-PAGE: sodium dodecyl sulfate polyacrylamide gel electrophoresis; KEGG: Kyoto Encyclopedia of Genes and Genomes; IPA: Ingenuity Pathway Analysis; GO: Gene Ontology; DEGs: Differentially expressed genes; IHC: immunohistochemistry; THOC2: THO complex subunit 2; TREX: Transcription-Export; PDE4D: Phosphodiesterase 4D; PIK3CA: phosphoinositide-3-kinase, catalytic, alpha polypeptide; GNAI1: G Protein Subunit Alpha I1; Adcy1: adenylyl cyclase type I; HHIP: Hedgehog interacting protein; ADORA2A: Adenosine A2a Receptor; GATA2: GATA binding protein 2; FOXA2: Forkhead box a2.

## Introduction

Melanoma is one of the most common types of malignant skin cancer worldwide [1]. Despite significant improvements in diagnostic techniques and treatments over the past few years, the 5-year survival rate for melanoma patients remains poor due to its proclivity to metastasize [2,3]. Therefore, the search for ideal and effective molecular markers of melanoma has been quite important in terms of improvement in the diagnosis and treatment of.

The THO/TREX (Transcription-Export) complex has been characterized as a multimeric protein complex that mediates transcription elongation (yeast), splicing of mRNAs (animals), and export of mRNAs from the nucleus [4–6]. THO complex subunit 2 (THOC2) depletion has been shown to interfere with mRNA export, chromosome alignment, mitotic progression, and genomic stability in humans [4,5] and to stimulate neurite outgrowth in primary rat hippocampal neurons [7]. Depletion of other TREX) subunits also interferes with mRNA export, resulting in nuclear retention of mRNAs Mouse Thoc2, along with Thoc5, is required for epithelial stem cell self-renewal and differentiation [8]. Thoc2 depletion in *Drosophila* Schneider 2 cells results in significant nuclear mRNA retention, inhibition of protein synthesis and cell proliferation, and chromosome misalignment [7,9]. Thoc2-knockout *Caenorhabditis elegans* are slow-growing, sterile, have functional defects in specific sensory neurons, and die prematurely from defective progression of meiosis [4,10]. Thoc2 is also an essential gene for *zebrafish* embryonic development [11]. However, the relationship between THOC2 and tumor progression remains unclear.

In this study, we determined the roles of THOC2 in melanoma progression. We found that THOC2 is up-regulated in melanoma at both the mRNA and protein levels. THOC2 silencing markedly reduced melanoma cell proliferation and invasion in vitro. Furthermore, we revealed that THOC2 might regulate cAMP signaling pathway in melanoma cells. Thus, we suggested that THOC2 might promote melanoma tumorigenesis via regulating the cAMP signaling pathway, which provides new insight into melanoma progression.

## Materials and methods

### Clinical specimens

Melanoma specimens and corresponding para-carcinoma tissues were collected from patients who underwent surgical treatment without preoperative radiotherapy and/or chemotherapy at the Chinese Academy of Medical Sciences and Peking Union Medical College. The specimens were fixed, prepared in paraffin blocks, and sectioned. All patients enrolled in the present study met the following criteria: (1) diagnosed with melanoma; (2) possessing complete clinicopathological data and follow-up information. The study was approved by the ethics committee at the Chinese Academy of Medical Sciences and Peking Union Medical College, and informed consent forms were signed by all patients.

### Cell culture

Human melanoma cell lines A-375 and M14 were purchased from the Cell Bank of Shanghai Institute of Cell Biology (Shanghai, China). Cells were maintained in RPMI-1640 medium, supplemented with 10% fetal bovine serum (FBS, Invitrogen, Carlsbad, USA), 1% penicillin/streptomycin (Sigma-Aldrich, Milan, Italy) at 37°C in 5% CO2.

### Cell transfection

siRNA against THOC2 (si-THOC2) and control siRNA (NC) were all purchased from IBSBIO (Shanghai, China). A375 and M14 cells were plated in 6-well plates at a density of 1.5 × 10^5^ per well. Cells were transfected with si-THOC2 or NC using Lipofectamine 2000 (Invitrogen) according to the manufacturer’s protocol. The sequences were as follows: NC 5′-UUCUCCGAACGUGUCACGUTT-3′,

THOC2 siRNA-1 5′- GAGUUGUCAUAUCAUGUAATT-3′,

THOC2 siRNA-2 5′-GCAGUACUUCUACAAUUUATT-3′,

THOC2 siRNA-3 5′-GCUAUGAACGAGAAGUCAATT-3′,

THOC2 siRNA-4 5′-GGUCAGAUCAGAAACACUATT-3′

### Reverse transcription quantitative real-time PCR(RT-qPCR)

Total RNA was isolated by TRIzol reagent (Invitrogen) according to the manufacturer’s instructions. cDNA was synthesized using a PrimeScript 1st strand cDNA synthesis kit (TaKaRaShuzo, Otsu, Japan). mRNA expression analysis was conducted by quantitative PCR using SYBR green dye(Life Technologies, Carlsbad, CA, USA). The expression levels were calculated using the 2^−ΔΔCt^ method with β-actin used for mRNA normalization . Primers used were as follows:

Actin-F, 5-CATGTACGTTGCTATCCAGGC-3;

Actin-R, 5-CTCCTTAATGTCACGCACGAT-3,

THOC2-F, 5-GGTAATCTTTCAGGAAGGTGGAGA-3; THOC2-R, 5-GCTGATGTCATCCCAGACTTTG-3.

PDE4D-F, 5-ACGGACCGGATAATGGAGGAG-3;

PDE4D-R, 5-ATTTTTCCACGGAAGCATTGTG-3.

PIK3CA-F, 5-CAGTCAGGAAAGGTGGTG −3;

PIK3CA-R, 5-ATTCAAAAGGTTCACGGA −3.

GNAI1-F, 5-TCTACAGTAACACCATCCAGTC −3;

GNAI1-R, 5-GCAGTCATAAAGCCTTCTTCAG-3.

ADCY1-F, 5-AGGCACGACAATGTGAGCATC-3;

ADCY1-R, 5- TTCATCGAACTTGCCGAAGAG-3.

HHIP-F, 5-CCCTGCATAGTGGGGATGG-3;

HHIP-R, 5- AGGCTTAGCAGTCCTCTTTCAT-3;

ADORA2A-R, 5-CTGCTGGCTGCCCCTACAC-3;

ADORA2A-F, 5- GAAGGGATTCACAACCGAATTG-3.

### Western blot assay

Proteins were lysed using RIPA buffer (Beyotime, Shanghai, China) and the concentrations were measured using a bicinchoninic acid Protein Assay Kit (CoWin, Beijing, China). Proteins were separated by 10% SDS-PAGE and transferred in PVDF membranes (Bio-Rad, Hercules, CA, USA). Following blocking with 5% low fat milk in Tris-buffered saline at 37°C for 1 h, the membranes were incubated with primary antibodies against THOC2 (1:1000, Abcam, USA), and β-actin (1:1000, Santa Cruz Biotechnology,Santa Cruz,CA, USA) overnight at 4°C. After incubation corresponding secondary antibodies at room temperature for 1 h, an electrochemiluminescence (ECL) system was used for detection.

### MTT assay

Transfected cells were dispensed in a 96-well plate (1500/per well) and incubated for 24, 48, 72 h, . 3-(4,5-dimethylthiazol-2-yl)-2,5-diphenyltetrazolium bromide (MTT) assays were performed by adding 20 μl of MTT for 4 h. Then, 150 μl of DMSO was added to each well. After 15 min, absorbance at 490 nm of each well was recorded by a microplate reader (Bio-Rad).

### Flow cytometric analysis

For apoptosis assays, transfected cells were harvested and marked with FITC-Annexin V and PI using and Annexin VFITC/PI kit(Life Technologies, Carlsbad, CA, USA). Utilizing FACS Calibur (BD Biosciences, San Jose, CA, USA), cell apoptosis was detected. The results were evaluated with FlowJo software.

### Transwell assay

Transfected cells (3 × 10^4^) were placed into upper chambers coated with 50 µl of Matrigel (8-μm-pore; Millipore, MA, USA). Medium supplemented with 10% FBS was added to the lower chamber. After 24 h of incubation, the cells from the upper surface were removed and the remaining cells on the lower surface were fixed in 4% paraformaldehyde and stained with 0.1% crystal violet for 30 min. The number of invading cells were measured under a microscope (Nikon, Tokyo, Japan).

### Immunohistochemistry (IHC)

IHC was performed using an HRP-polymer anti-mouse IHC DAB-based kit (IBSBIO, Shanghai, China) according to the manufacturer’s protocol and a previous study [12].

### Microarray analysis

Total RNA samples were sent out to IBSBIO Company (Shanghai, China) and then applied to Agilent SurePrint G3 Human Gene Expression v3 Microarray (8*60K,Design ID:072363) to assess mRNA expression profiles. The sample labeling, microarray hybridization and washing were performed based on the manufacturer’s standard protocols. Briefly, total RNA was transcribed to double stranded cDNA and then cRNA was synthesized . Next, 2nd cycle cDNAs were synthesized from cRNAs. Following fragmentation and biotin labeling, the 2nd cycle cDNAs were hybridized to the microarray. After washing and staining, the arrays were scanned using an Agilent G2505C array scnner(Agilent Technologies).

Feature Extraction software (Agilent Technologies) was used to extract raw data and provide RNA normalization. Genesrping software (Agilent Technologies) was employed to finish the basic analysis. Differentially expressed genes (DEGs) were then identified through fold change as well as P value calculated with Student’s t-test. The threshold set for up- and down-regulated genes was fold change ≥ 2.0 and a P value ≤ 0.05.

### Bioinformatics analysis

Oncomine gene expression array database (www.oncomine.org) was used to explore *THOR2* mRNA levels in melanoma tissues. ALTAS dataset was used to explore effects involving the prognosis of patients. Gene Ontology (GO) and KEGG analyze were applied to determine the roles played differentially expressed mRNAs in these GO terms or pathways. Finally, Hierarchical Clustering was performed to display distinguishable gene expression patterns among samples. Ingenuity Pathway Analysis (IPA) expression network was used to predict downstream effects. The interaction pathway analysis was performed using the STRING database (http://string-db.org/). Microarray files have been deposited in the GEO database with accession number GSE109497.

### Statistical analysis

Statistical analysis was done using SPSS 20.0. Data are shown as means ± standard deviation (SD) and each experiment was repeated three times. Paired and unpaired Student’s t-tests were used to analyze data between two groups of samples, whereas comparisons among multiple groups were done by ANOVA followed by Tukey’s post hoc test. P < 0.05 was considered statistically significant.

## Results

### Elevated THOC2 expression in melanoma

**Figure 1.**
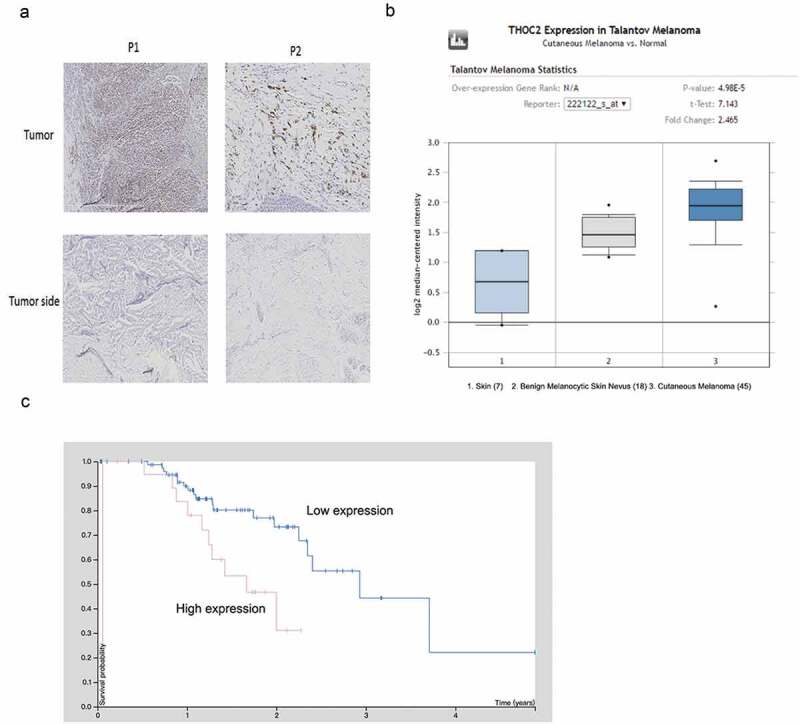
THOC2 was upregulated in melanoma.(a) IHC staining for THOC2 in melanoma tissues and adjacent normal tissues. (b) Oncomine expression analysis of THOC2 in human melanoma vs. normal skin tissues and benign melanocytic skin nevus. (c) Melanoma patients with high THOC2 expression had a poor prognosis compared to thelow THOC2 expression group. *P < 0.05.

To examine THOC2 expression in human melanoma, IHC was used. Results showed that THOC2 was mainly stained in the nuclei of melanoma cells and upregulated in melanoma tissues compared to normal skin tissue (Figure 1(a)). Subsequently, THOC2 expression was explored in Oncomine database, Results showed that THOC2 is highly expressed using the melanoma tissues (Figure 1(b)). Furthermore, Kaplan-Meier survival analysis showed that melanoma patients with high THOC2 expression had a poor overall survival rate compared to the low expression group (Fi.1C). These data suggested that THOC2 might play critical roles in melanoma progression.

### THOC2 inhibition reduced melanoma cells proliferation

**Figure 2.**
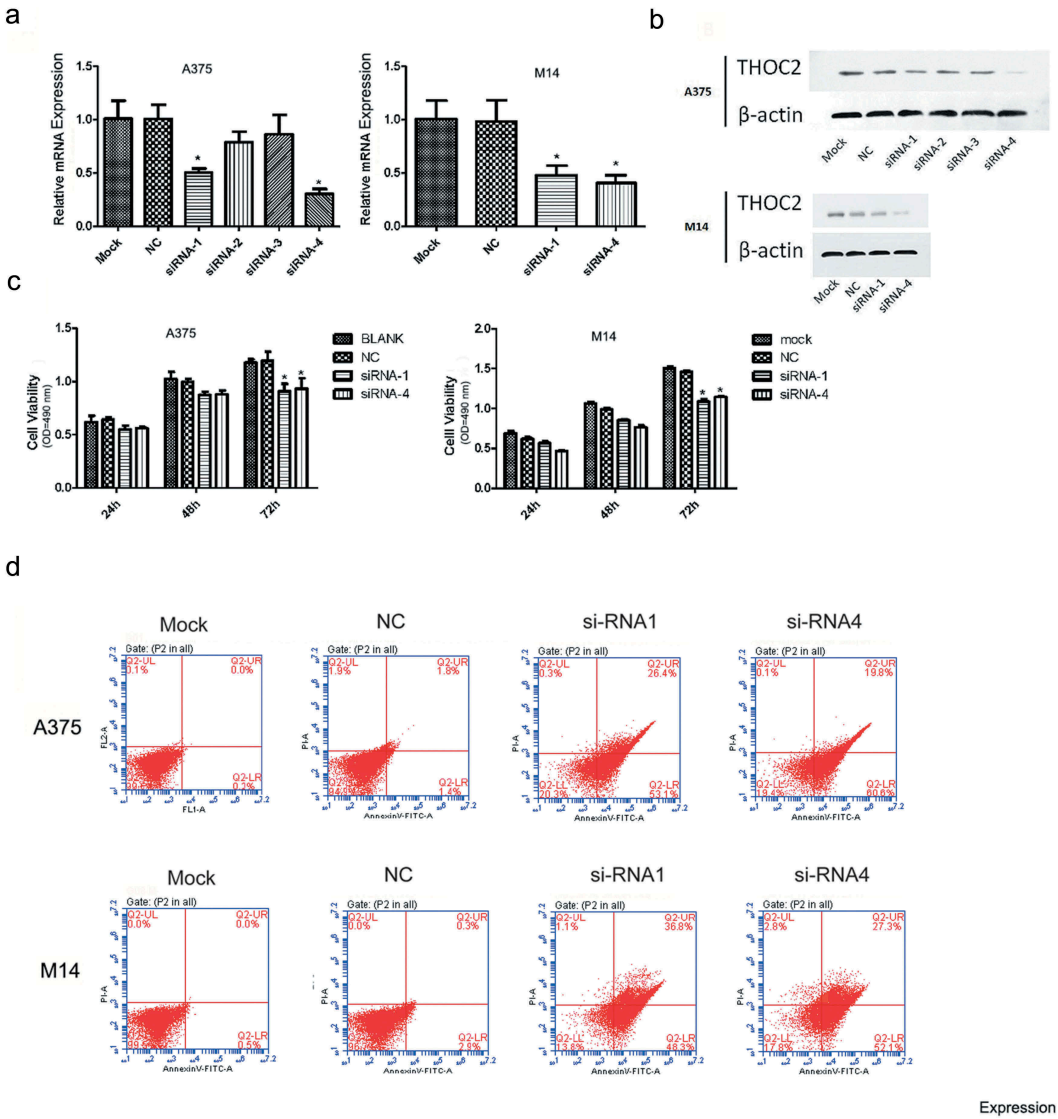
THOC2 inhibition reduced melanoma cell proliferation. (a, b) Relative expression of THOC2 in A375 and M14 cells transfected with siRNAs or NC was measured by RT-qPCR and western blot. (c) MTT assays showed that THOC2 inhibition reduced melanoma cell proliferative ability in vitro. (d) Flow cytometric analysis showed that THOC2 inhibition increased apoptosis of melanoma cells. *P < 0.05.

Next, we examined the roles of THOC2 in melanoma. siRNAs targeting THOC2 were transfected into A375 and M14 cells. Transfection efficiency was determined by RT-qPCR and western blot (Figure 2(a,b)). Subsequently, MTT assays showed that THOC2 inhibition decreased the proliferation of A375 and M14 cells in vitro (Figure 2(c)). Flow cytometry assays showed that knockdown of THOC2 dramatically increased the apoptosis rate of A375 and M14 cells (Figure 2(d)). These data suggested that THOC2 could promote melanoma cells proliferation and restrain cell apoptosis in vitro.

### Depletion of THOC2 suppressed melanoma cell invasion

**Figure 3.**
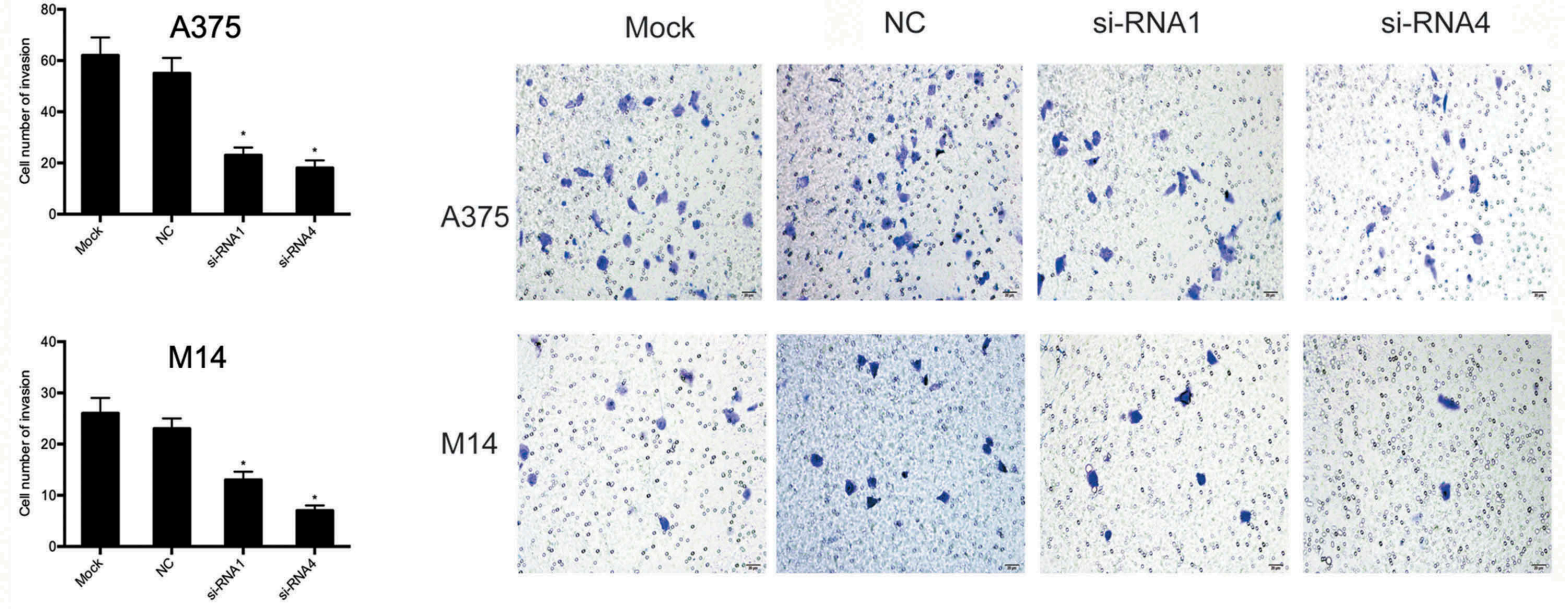
THOC2 inhibition reduced melanoma cells invasion. *P < 0.05.

Next, we investigated the effects of THOC2 on melanoma cell invasion. Transwell assays showed that THOC2 suppression significantly suppressed the invasive ability of A375 and M14 cells compared to the NC group (Figure 3).

### Identification of THOC2-dependent genes in melanoma cells

**Figure 4.**
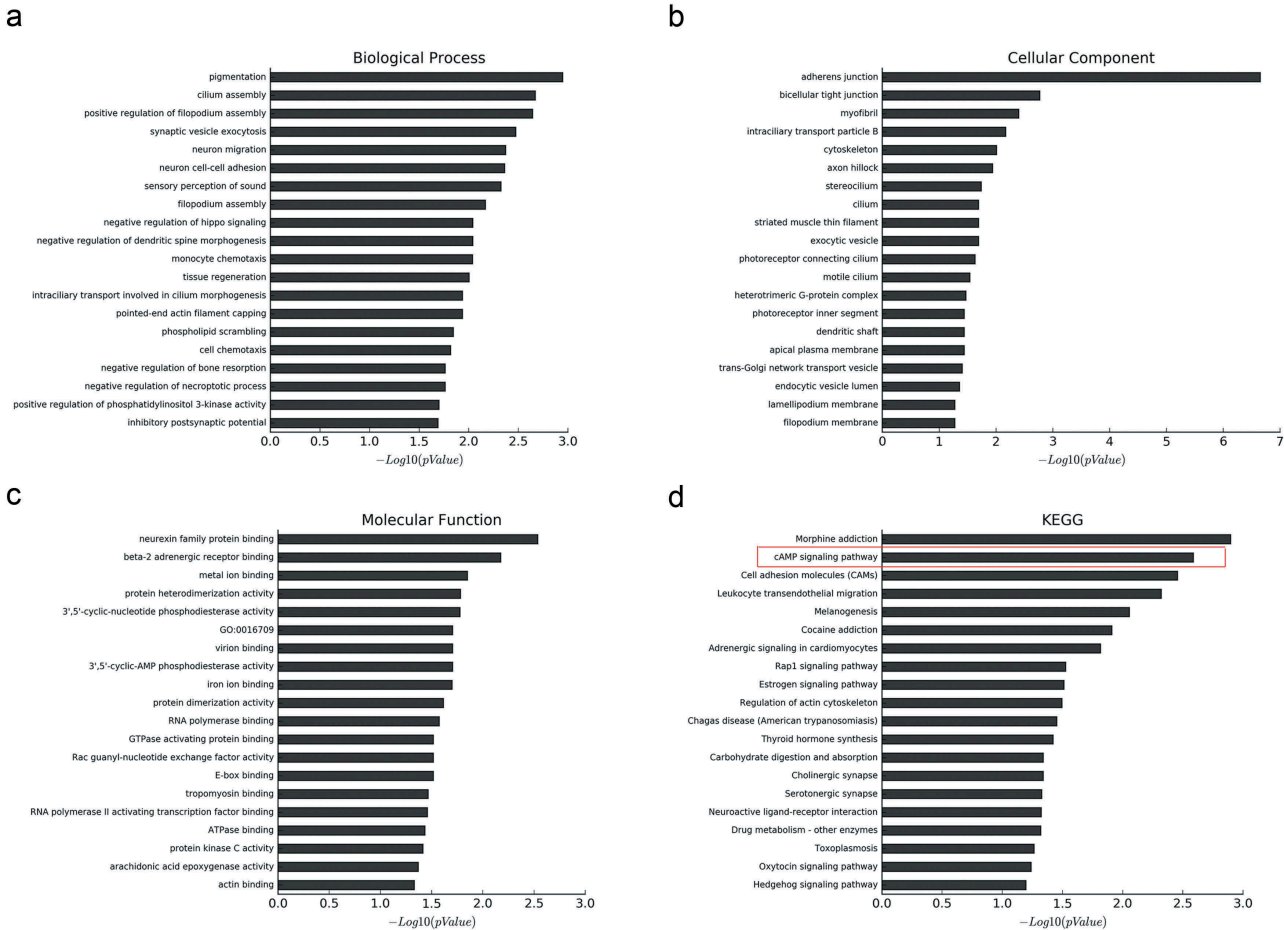
Downregulation of genes upon knockdown of THOC2 in A375 cells. (a) The most frequent fold enrichment biological processes (BP) associated with downregulated coding gene transcripts. (b) The most frequent fold enrichment cellular components (CC) associated with downregulated coding gene transcripts. (c) The most frequent fold enrichment molecular functions (MF) associated with downregulated coding gene transcripts. (d) KEGG analysis of downregulated coding gene transcripts.

In our study, A375 cells were transfected with si-THOC2 and si-NC, and mRNA from A375 cells wase collected for microarray analysis. A total of 877 genes were downregulated more than 2-fold upon the depletion of THOC2 in A375 cells. To investigate the molecular functions and biologic pathways involving THOR2, all 877 downregulated genes were further analyzed by Gene Ontology (GO) and KEGG analyses. GO enrichment analysis showed that THOR2 might play important roles in cellular biological functions (Figure 4(a–c)). KEEG pathway analysis showed that the cAMP signaling pathway is one of the most closely signaling pathways correlated with THOR2 in melanoma progression (Figure 4(d)). To further determine the biological relevance of the above-identified down-regulated mRNAs, IPA was used. Disease and function analysis showed that the majority of the downregulated mRNAs were associated with cancer progression (Table 1).

**Table 1.** Down-regulated coding gene transcripts corresponding to top 10 disease and functions.

Diseases and functions	p-value	Number of molecular
Cancer	1.79E-10	343
Organismal Injury and Abnormalities	1.79E-10	344
Gastrointestinal Disease	6.65E-07	289
Hepatic System Disease	6.65E-07	165
Dermatological Diseases and Conditions	6.36E-06	207
Reproductive System Disease	1.17E-05	191
Tissue Morphology	2.40E-05	33
Cellular Assembly and Organization	3.85E-05	82
Hair and Skin Development and Function	3.85E-05	4
Cellular Function and Maintenance	8.82E-05	80

### THOC2 regulated camp pathway in melanoma cells

**Figure 5.**
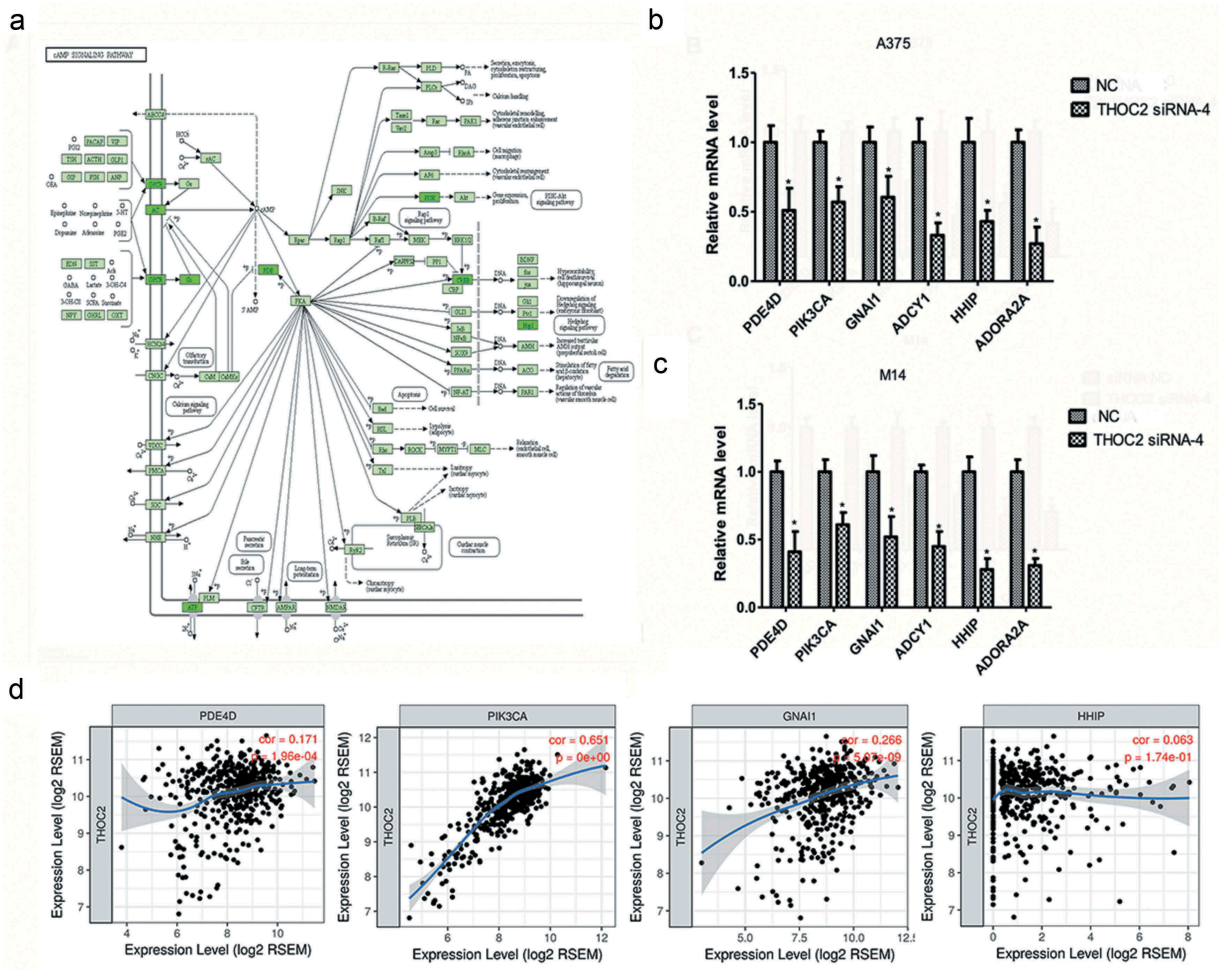
THOC2 regulated cAMP pathway in melanoma cells. (a) cAMP signaling pathway. (b, c) THOC2 inhibition reduced cAMP pathway related gene expression (*PDE4D, PIK3CA, GNAI1, ADCY1, HHIP* and *ADORA2A*) in A375 cells (b) and M14 cells (c). (d) THOC2 expression was positively correlated with *PDE4D, PIK3CA, GNAI1*, and *HHI*P expression in melanoma tissues. *P < 0.05.

The cAMP signaling pathway (Figure 5(a)) has been shown to play critical roles in tumor progression, including that of melanoma progression. Our previous study showed that THOC2 might associate with the cAMP signaling pathway in melanoma progression. Thus, we further explored cAMP pathway-related gene expression by RT-qPCR in melanoma cells. Results showed that the expression levels of *PDE4D, PIK3CA, GNAI1, ADCY1, HHIP* and *ADORA2A* were significantly reduced in A375 and M14 cells transfected with si-*THOC2* compared to theNC group (Figure 5(b,c)). In addition, correlation analysis showed that THOC2 expression was correlated with *PDE4D, PIK3CA, GNAI1*, and *HHIP* expression in melanoma tissues (Figure 5(d)). The data further confirmed that THOC2 might regulate he cAMP signaling pathway in melanoma cells.

### THOC2 network analysis in melanoma

**Figure 6.**
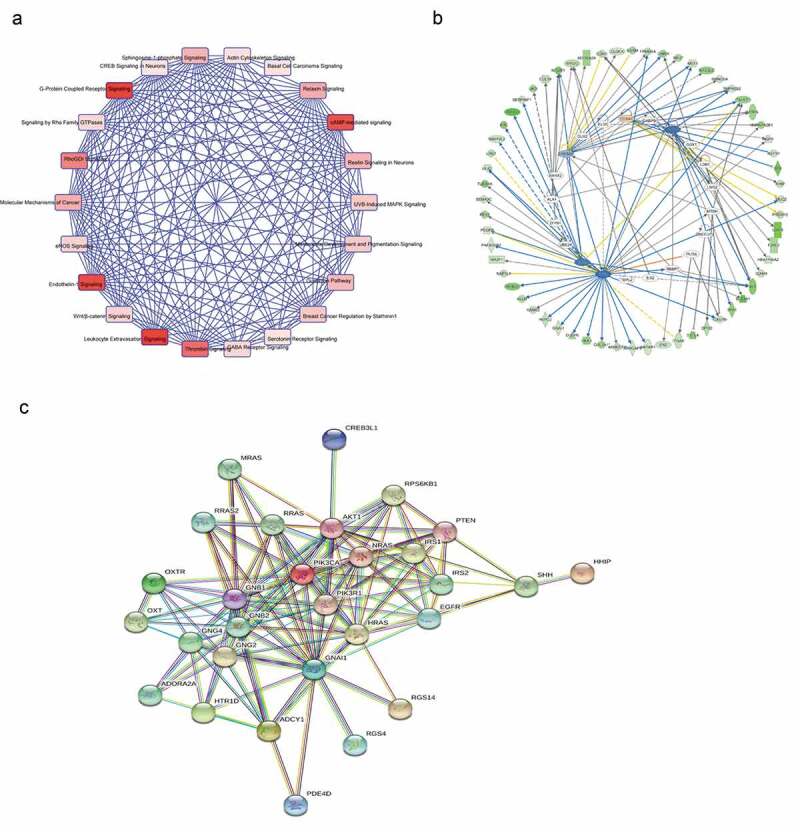
THOC2 network analysis in melanoma. (a) IPA expression network in melanoma. (b) IPA analysis of regulated proteins. (c) STRING program was used to exporehe protein-protein interactions involving THOC2 and cAMP pathway-related genes.

Next, we performedIPA expression network in melanoma (Figure 6(a)). Candidate genes of canonical pathway involved in cAMP signaling pathway. We carried out Ingenuity Upstream Regulator Analysis in IPA to identify the cascade of upstream transcriptional regulators that can explain the observed changes in proteins. As predicted by IPA, the proteinsregulated in our study are likely to be modulated by GATA2, TP53, TP73 and FOXA2 (Figure 6(b)). Then, we performed interaction pathway analysis using the STRING database (http://string-db.org/). Results identified potentially regulated interacting proteins involving the cAMP pathway and the proteins involved in cell proliferation and metastasis (Figure 6(c)). Thus, we inferred that THOC2 might play important roles in melanoma progression by regulating the cAMP pathway.

## Discussion

Melanoma is derived from neural crest melanocytes and is frequently found in the skin, digestive tract, eyes, genitals, and nasal cavity [13,14]. Due to distant metastases, the poor prognosis of melanoma patientsremans poor [15]. In this study, we examined the expression and the clinical relevance of THOC2 in melanoma. We showed that THOC2 expression was significantly increased in melanoma tissues. High THOC2 expression was associated with poor prognosis of patients. In functional assays, ourresults showed that THOC2 suppression reduced the proliferation and invasion of melanoma cells. Moreover, down-regulated THOC2 promoted melanoma cell apoptosis. These data suggested that THOC2 acts as a novel target for the treatment of melanoma.

Recent studies showed that increased cAMP signaling could promote melanoma tumor progression. For example, Kim et al found that induction of ATP synthase β by H_2_O_2_ induces melanogenesis by activating PAH and cAMP/CREB/MITF signaling in melanoma cells [16]. Jun et al found that phloridzin-induced melanogenesis is mediated by the cAMP signaling pathway [17]. Ma et al revealed that miR-23a-3p inhibits mucosal melanoma growth and progression through targeting adenylate cyclase 1 and attenuating the cAMP and MAPK pathways [18]. However, the roles of the cAMP pathway in melanoma remain unclear.

mRNA processing, splicing, and export are tightly regulated by a complex RNA-protein network which is essential for the maintenance of cellular and tissue homeostasis [19]. Depletion of one of the RNA processing regulators influences the expression of a set of genes. It is possible that these regulators might serve as useful target molecules for cancer therapy [20,21]. Recent studies showed that THOC2 depletion interferes with mRNA export, chromosome alignment, mitotic progression, and genomic stability in humans [4,5]. Thus, we speculated that THOC2 plays an important role in tumor development. From our data, GO and KEGG analysis showed that THOR2 might play important roles in cellular biological functions, and be associated with the cAMP signaling pathway in melanoma progression.

Next, we explored cAMP pathway related gene expression in THOC2 inhibited melanoma cells. Our results showed that THOC2 inhibition significantly reduced the expression of *PDE4D, PIK3CA, GNAI1, ADCY1, HHIP* and *ADORA2A* in melanoma cells. THOC2 expression was positively correlated with *PDE4D, PIK3CA, GNAI1*, and *HHI*P expression in melanoma tissues. Subsequently, IPA expression network investigation was used to identify the cascade of upstream transcriptional regulators. As predicted by IPA, the proteins regulated in our study are likely to be modulated by GATA2, TP53, TP73 and FOXA2. Furthermore, STRING database analysis showed that THOC2 might play roles in melanoma cells by interacting with proteins in the cAMP pathway and with proteins involved in cell proliferation and metastatic processes.

In conclusion, we demonstrated that THOC2 was significantly up-regulated in melanoma. THOC2 suppression reduced the proliferation of invasion by melanoma cells in vitro, at least partly by regulating the cAMP pathway. These findings suggested that THOC2 might could be used as a new predictive biomarker and therapeutic target in melanomapatients.

## Data Availability

The datasets supporting the conclusions of this article are included within the article.

## Highlights

- THOC2 is upregulated in melanoma.
- THOC2-cAMP pathway regulats melanoma progression.
- THOC2 might serve as an important therapeutic target for melanoma treatment.

## References

[1] Cummins DL, Cummins JM, Pantle H, et al Cutaneous malignant melanoma[C]//Mayo clinic proceedings. Elsevier. 2006;81(4):500–507. 10.4065/81.4.500 16610570

[2] Hodi FS, O’Day SJ, McDermott DF, et al Improved survival with ipilimumab in patients with metastatic melanoma. N Engl J Med. 2010;363(8):711–723. 20525992 10.1056/NEJMoa1003466 PMC3549297

[3] Garbe C, Leiter U. Melanoma epidemiology and trends. Clinics Dermatol. 2009;27(1):3–9. 10.1016/j.clindermatol.2008.09.001 19095149

[4] Di Gregorio E, Bianchi FT, Schiavi A, et al A de novo X; 8 translocation creates a PTK2-THOC2 gene fusion with THOC2 expression knockdown in a patient with psychomotor retardation and congenital cerebellar hypoplasia. J Med Genet. 2013;50(8):543–551. 23749989 10.1136/jmedgenet-2013-101542 PMC4133931

[5] Francisco-Mangilet AG, Karlsson P, Kim MH, et al THO 2, a core member of the THO/TREX complex, is required for micro RNA production in Arabidopsis. Plant J. 2015;82(6):1018–1029. 25976549 10.1111/tpj.12874

[6] Yamazaki T, Fujiwara N, Yukinaga H, et al The closely related RNA helicases, UAP56 and URH49, preferentially form distinct mRNA export machineries and coordinately regulate mitotic progression. Mol Biol Cell. 2010;21(16):2953–2965. 20573985 10.1091/mbc.E09-10-0913 PMC2921121

[7] Rehwinkel J, Herold A, Gari K, et al Genome-wide analysis of mRNAs regulated by the THO complex in Drosophila melanogaster. Nat Struct Mol Biol. 2004;11(6):558–566. 15133499 10.1038/nsmb759

[8] Wang L, Miao YL, Zheng X, et al The THO complex regulates pluripotency gene mRNA export and controls embryonic stem cell self-renewal and somatic cell reprogramming. Cell Stem Cell. 2013;13(6):676–690. 24315442 10.1016/j.stem.2013.10.008 PMC3962795

[9] Somma MP, Ceprani F, Bucciarelli E, et al Identification of Drosophila mitotic genes by combining co-expression analysis and RNA interference. PLos Genet. 2008;4(7):e1000126. 18797514 10.1371/journal.pgen.1000126 PMC2537813

[10] Castellano-Pozo M, García-Muse T, Aguilera A R-loops cause replication impairment and genome instability during meiosis. EMBO Rep. 2012;13(10):923–929. 22878416 10.1038/embor.2012.119 PMC3463965

[11] Amsterdam A, Nissen RM, Sun Z, et al Identification of 315 genes essential for early zebrafish development. Proc Natl Acad Sci USA. 2004;101(35):12792–12797. 15256591 10.1073/pnas.0403929101 PMC516474

[12] Yan Y, Yang F, Zhang H, et al Up-regulation of flotillin-2 is associated with renal cell carcinoma progression. Tumor Biol. 2014;35(10):10479–10486. 10.1007/s13277-014-2343-9 25053596

[13] Bertolotto C Melanoma: from melanocyte to genetic alterations and clinical options. Scientifica (Cairo). 2013;2013:635203. 24416617 10.1155/2013/635203 PMC3874946

[14] Agaimy A, Specht K, Stoehr R, et al Metastatic malignant melanoma with complete loss of differentiation markers (undifferentiated/dedifferentiated melanoma). Am J Surg Pathol. 2016;40(2):181–191. 26448190 10.1097/PAS.0000000000000527

[15] Allen DC Malignant melanoma[M]//Histopathology reporting. London: Springer; 2013 p. 207–216.

[16] Kim HE, Lee SG Induction of ATP synthase ß by H2O2 induces melanogenesis by activating PAH and cAMP/CREB/MITF signaling in melanoma cells. Int J Biochem Cell Biol. 2013;45(7):1217–1222. 23523934 10.1016/j.biocel.2013.03.006

[17] Jung E, Lee J, Huh S, et al Phloridzin-induced melanogenesis is mediated by the cAMP signaling pathway. Food Chem Toxicol. 2009;47(10):2436–2440. 19576939 10.1016/j.fct.2009.06.039

[18] Ma M, Dai J, Tang H, et al MicroRNA-23a-3p inhibits mucosal melanoma growth and progression through targeting adenylate cyclase 1 and attenuating cAMP and MAPK pathways. Theranostics. 2019;9(4):945. 30867808 10.7150/thno.30516 PMC6401396

[19] Wickramasinghe VO, Laskey RA Control of mammalian gene expression by selective mRNA export. Nat Rev Mol Cell Biol. 2015;16(7):431–442. 26081607 10.1038/nrm4010

[20] Hayes J, Peruzzi PP, Lawler S MicroRNAs in cancer: biomarkers, functions and therapy. Trends Mol Med. 2014;20(8):460–469. 25027972 10.1016/j.molmed.2014.06.005

[21] Garzon R, Marcucci G, Croce CM Targeting microRNAs in cancer: rationale, strategies and challenges. Nat Rev Drug Discovery. 2010;9(10):775. 20885409 10.1038/nrd3179 PMC3904431

